# 

**Published:** 1851-01

**Authors:** 


### NOTICE TO READERS AND CORRESPONDENTS.

We have received original communications from Drs. W. W. Goff, James Smick, D. Hutchinson, and J. W. Spalding, which shall be attended to in due time.

Dr. Underwood of Peoria, some time ago requested as to resume our abstracts of the Journal at the close of the volume. We shall try to do so in our next.

We have received for notice The Physician and Patient, and History of Medical Delusions. By Worthington Hooker, M. D., from Messrs. Burley of Chicago.

Review of Chemistry for students. By J. G. Murphy, M. D., from Lindsay and Blakiston.

Introductory Lecture of Prof. Curran, to the class in the Indiana Central Medical College.

Introductory address by Prof. Pallen, of St. Louis University.

Disease of the Kidney, by F. A. Ramsey, A. M., M. D.

The following Journals are regularly received.

New Hampshire Journal of Medicine.
Boston Medical and Surgical Journal.
New York Journal of Medicine and Collateral Sciences.
New York Register of Medicine and Pharmacy.
New York Medical Gazette.
Braithwaites Retrospect.
London Lancet.
British and Foreign Medico Chirurgical Review.
American Journal of Medical Sciences.
Medical News.
Medical Examiner.
Transactions of Philadelphia College of Physicians.
Ranking's Abstract.
American Journal of Pharmacy.
New Jersey Medical Reporter.
American Journal of Dental Science.
Charleston Medical Journal and Review.
Southern Medical and Surgical Journal.
New Orleans Medical and Surgical Journal.
St. Louis Medical and Surgical Journal.
The Probe.
Western Medico Chirurgical Journal.
Western Journal of Medicine and Surgery.
Transylvania Medical Journal.
Western Lancet and Hospital Reporter.
Ohio Medical and Surgical Journal.
Buffalo Medical Journal.
Northern Lancet and Gazette of Legal Medicine.

### NOTICE TO SUBSCRIBERS.

The time during which this volume may be paid for, with *two* dollars, has nearly expired. One number more closes the year, and our terms are, that those who do not pay during the year will be charged *three dollars*. Pray use a little more economical promptness and save one dollar by paying up before the close of the year.

### LIST OF RECEIPTS FOR NOVEMBER AND DEGEMBER.

### MICHGAN.

| | |
|---|---|
| Dr. Hiram Sheldon, | $3,0[0] |
| R. Stephenson, | 1,00 |
| Jesse Wasson, | 2,00 |
| Edwin Stewart, | 2,00 |
| W. B. Lincoln | 2 00 |
| B. Bingham, | 6,00 |
| Isaac Snyder, | 2,00 |
| E. Lewis, | 2,00 |
| J. McLean, | 2.00 |
| H. Davis, | 2,00 |
| John Acers, | 2.00 |
| T. S. Tuttle, | 2,00 |
| —Peck, | 2,00 |
| J. *S*. Skinner, | 2,00 |
| L. Foster | 5.00 |
| R. S. *H*awley, | 2,00 |

### INDIANA,

| | |
|---|---|
| Dr. W Matthews, | 2,00 |
| T. W Meller, | 2,00 |
| J. E. Hendricks, | 2,00 |
| J. P. Tucks, | 2,00 |
| McMechan, | 6,00 |
| J. F. Parker, | 2.00 |
| Jno. Lewis, | 2,00 |
| E. R. Ford, | 2,00 |
| W. F. Boor, | 2.00 |
| R. Willard, | 2,00 |
| L. D. Hogle, | 2,00 |
| J. P. Russell, | 4,00 |
| U. A- V. *H*ester, | 1,00 |
| M. Chiles, | 75 |
| Clay Brown, | 2,00 |
| D. W, *S*kinner, | 2,00 |
| W. T. Green, | 4,00 |
| M. Bell, | 2,00 |

### WISCONSIN.

| | |
|---|---|
| Dr. D C Ayres | 2,00 |
| W Lewis | 2.00 |
| R W Earll | 2,00 |
| H. McKinley | 2,00 |
| B Salesbury | 2.00 |
| *B* F Collins | 2,00 |
| J. Keenen | 2,00 |
| O *D*. Coleman | 2,00 |
| M R Fowler | 6,00 |
| Jno. Parker | 2,00 |
| I P Hamilton | 2.00 |
| J S Russell | 2.00 |
| R T Hayes | 2.00 |
| Jos. Pasko | 2,00 |

### ILLINOIS.

| | |
|---|---|
| Dr. L W Warren | 2.00 |
| J F Spilman | 5,00 |
| B F Stephenson | 2,00 |
| W W R Woodbury | 2.00 |
| *W W* Sedgwick | 2,00 |
| *W*m Robinson | 2 00 |
| V A Boyer | 10,00 |
| T G Klepper | 2,00 |
| L F Nelson | 2,00 |
| E Friend | 2,00 |
| N B Judd, Esq. | 4'00 |
| Dr. *R* McGee | 2.00 |
| M F Irwin | 4 00 |
| O S Johnson | 2,00 |
| *W* H Winter | 2,00 |
| Hugh Martin | 2,00 |
| John Warner | 4'00 |
| Z H Whitmire | 4,25 |
| E C Banks | 2,00 |
| Thos Wilkins | 2,00 |
| A L Kellar | 2,00 |
| J G Tilden | 2,00 |
| W H Snyder | 2,00 |
| F B Ives | 5,00 |
| J S King | 2,50 |
| W H Crandall | 4.00 |
| H S Hall | 4.00 |
| J G Harlan | 3,00 |
| J A Halderman | 2,00 |
| C Goodbrake | 2,00 |
| S H Washburn | 2,00 |
| *P* Reinbold | 2.00 |
| W F Hutchinson | 4,00 |
| Josiah Burritt | 2,00 |
| A Mahan | 4,00 |
| A L McArthur | 2.00 |
| L Avery | 2.00 |

### NEW YORK.

| | |
|---|---|
| Dr. R Scott | 1,00 |

### OHIO.

| | |
|---|---|
| Dr. S Stough | 2·00 |
| R H Timpany | 2.00 |
| G N Bentler | 2,00 |
| A H Tyler | 2,00 |

### MINNESOTA.

| | |
|---|---|
| Dr. John H Murphy | 4,25 |

### IOWA.

| | |
|---|---|
| Dr. J M Weatherwax | 2,0[0] |
| J F Henry | 2,0[0] |
| W H Rousseau | 2,00 |

